# GPX1, a biomarker for the diagnosis and prognosis of kidney cancer, promotes the progression of kidney cancer

**DOI:** 10.18632/aging.102555

**Published:** 2019-12-16

**Authors:** Yongbiao Cheng, Tianbo Xu, Sen Li, Hailong Ruan

**Affiliations:** 1 Department of Urology, Union Hospital, Tongji Medical College, Huazhong University of Science and Technology, Wuhan 430022, China; 2 Institute of Urology, Union Hospital, Tongji Medical College, Huazhong University of Science and Technology, Wuhan 430022, China

**Keywords:** GPX1, kidney cancer, diagnosis, prognosis, biomarker, progression

## Abstract

Renal cell carcinoma (RCC) is the most common malignant tumor of the kidney, and its diagnosis and prognosis still lack reliable biomarkers. Glutathione peroxidase 1 (GPX1) has been identified to be highly expressed in a variety of human malignancies. However, few studies have studied the expression of GPX1 and its biological functions in RCC. We attempted to assess the potential of GPX1 as a promising biomarker for RCC diagnosis and prognosis. In this study, we analyzed and explored the public cancer databases (TCGA and ONCOMINE) to conclude that GPX1 is highly expressed in RCC. Meanwhile, we evaluated the expression of GPX1 at the levels of RCC cells and tissues to verify the results of the database. Moreover, high GPX1 levels were positively correlated with short overall survival time, distant metastasis, lymphatic metastasis, and tumor stage. Receiver operating characteristic curve (ROC) analysis showed that high GPX1 levels could distinguish RCC patients from normal subjects (p < 0.0001). Kaplan-Meier curve analysis revealed that high GPX1 levels predicted shorter overall survival time (p = 0.0009). Finally, the functional roles of GPX1 were examined using a GPX1 sh-RNA knockdown method in RCC cell lines. In summary, our results suggest that GPX1 may have the potential to serve as a diagnostic and prognostic biomarker for RCC patients. Moreover, targeting GPX1 may represent as a new therapeutic strategy and direction for RCC patients.

## INTRODUCTION

Renal cell carcinoma (RCC) is the most common malignant tumor of the kidney, which accounts for about 80-90% of kidney malignancies and approximately 2-3% of systemic malignancies. It is estimated that there are approximately 73,820 new cases of kidney cancer and a predicted 14,770 deaths in the United States in 2019 [1]. According to the WHO classification criteria, RCC consists of multiple pathological subtypes. Among all RCC pathological subtypes, clear cell renal cell carcinoma (ccRCC) is the most common pathological subtype, usually accompanied by high metastasis rate and high mortality, and is not sensitive to radiotherapy and chemotherapy. In recent years, although great progress has been made in the study of tyrosine kinase inhibitors and immune checkpoint inhibitors, many advanced or metastatic patients die of RCC due to insensitivity or tolerance to these drugs [2, 3]. Early diagnosis and timely surgical treatment are still key factors in the treatment of localized RCC. However, due to the lack of reliable and specific diagnostic biomarkers, approximately 15% RCC patients have progressed into distant metastasis at clinical diagnosis, resulting in poor prognosis [4]. Therefore, there is an urgent need to find RCC-specific diagnostic biomarkers and new therapeutic targets, and look forward to improving the early diagnosis rate of RCC and the cure rate of metastatic RCC.

Reactive oxygen species (ROS), such as hydrogen peroxide, superoxide and hydroxyl radicals, are produced in all cells by enzymatic and mitochondrial sources [5]. ROS are continuously produced in and cleared from cells through a series of complicated synthesis and degradation pathways [6]. When the balance of synthesis and degradation is broken, ROS can cause oxidative damage to proteins, DNA and membrane unsaturated fatty acids. Tumor cells produce more ROS than normal cells due to stronger metabolism and relative hypoxia-induced mitochondrial dysfunction [7]. It is well known that excessive ROS can cause apoptosis of tumor cells. Nevertheless, excessive ROS levels in tumor cells are counteracted by antioxidant enzyme-catalyzed reduction reactions to avoid the adverse effects of oxidative stress [8, 9]. The antioxidant enzyme system is composed of superoxide dismutase, thioredoxin peroxidase, glutathione peroxidase, catalase and others. In mammals, the glutathione peroxidases (GPXs) family consists of eight members (GPX1-GPX8) identified so far; five of them (GPX1-4 and GPX6) contain selenocysteine in the catalytic center and the other three are cysteine-containing proteins.

GPX1, diffusely distributed in the cytoplasm and mitochondria [10], is one of the most critical members of the GPXs family that catalytically reduces hydrogen peroxide to produce water [5]. GPX1 has been reported to be involved in both pro- and anticancer effects in different tumor models. Such as, the high expression of GPX1 was significantly associated with nodal metastasis, high grade, depth of tumor invasion, perineural invasion and advanced overall stage, and predicts poor prognosis in oral squamous cell carcinoma [11]. In a mouse model of skin cancer, overexpression of GPX1 increased the number of tumors and promotes their growth [12]. In contrast, GPX1 overexpression inhibited the growth of pancreatic cancer cells in vitro and in vivo models [13]. In addition, GPX1 knockdown in prostate cancer cells could enhance radiation-induced micronuclei formation [14]. In summary, GPX1 plays a different role in different tumor models. However, only few studies have explored the expression levels of GPX1 and its biological functions in ccRCC. Therefore, our aim is to study the expression level of GPX1 and its potential for diagnosis and prognosis of ccRCC.

## RESULTS

### GPX1 is significantly high expression and correlated with a range of clinical pathological parameters in ccRCC tissues

**Figure 1:**
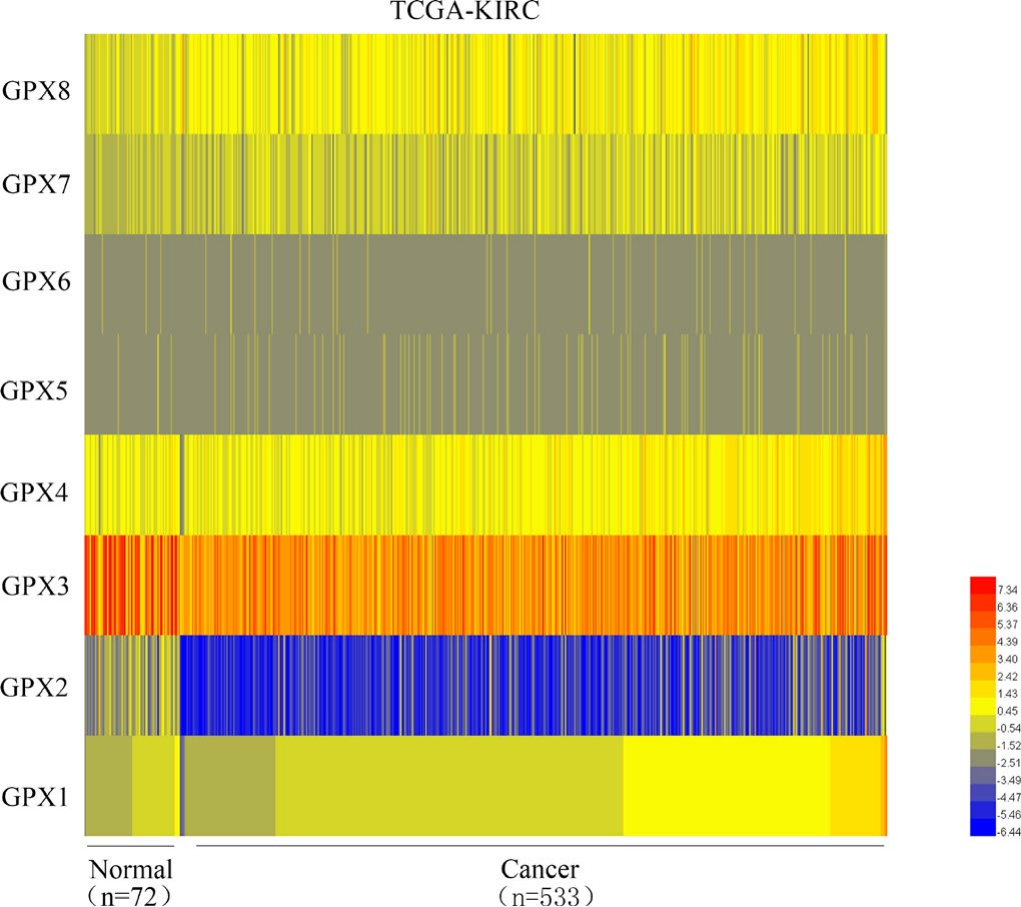
**Heat map depicts the expression of GPXs family members in ccRCC samples from the TCGA-KIRC database (n=605).** Red signifies high expression levels, blue signifies low expression levels, and yellow signifies medium expression levels. TCGA: The Cancer Genome Atlas; KIRC: kidney renal clear cell carcinoma.

**Figure 2:**
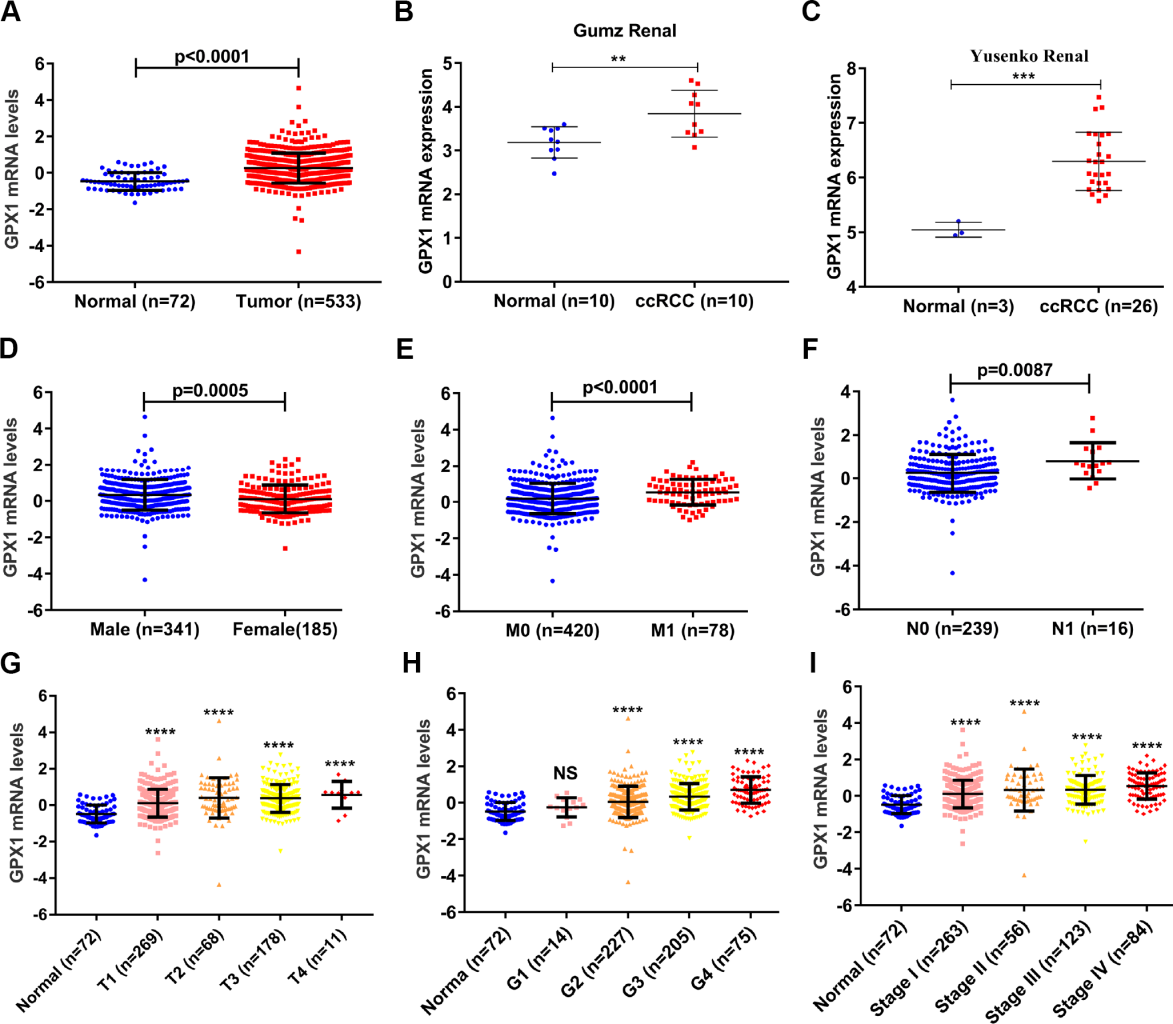
**GPX1 is overexpressed and positively associated with higher tumor stage in ccRCC samples.** (**A**) The mRNA expression levels of GPX1 were up-regulated in ccRCC samples, which were downloaded from TCGA-KIRC database containing 72 normal samples and 533 ccRCC samples. (**B**–**C**) The mRNA expression levels of GPX1 were up-regulated in ccRCC in statistics by Gumz et al. and Yusenko et al, which were downloaded from ONCOMINE database. The expression levels of GPX1 mRNA were compared in various clinical pathological parameters: (**D**) Gender, (**E**) M stage, (**F**) N stage, (**G**) T stage, (**H**) G stage, (**I**) TNM stage. (T means Territory; M means distant metastasis; N means Lymph node metastasis. ****, P < 0.0001, ***, P < 0.001, **, P < 0.01, NS means no significance, compared with the respective control).

As mentioned above, the GPXs family has 8 members. We used the TCGA database to mine the expression levels of 8 members in ccRCC and found that GPX1 was significantly up-regulated in ccRCC (n=533) compared with adjacent normal renal tissues (n=72) (Figure 1), so we chose GPX1 as the research target. To quantify the expression levels of GPX1, the TCGA and ONCOMINE databases were used to mine the sequencing data of GPX1 in ccRCC. As shown in Figure 2A, the expression levels of GPX1 were significantly higher in ccRCC tissues than that in adjacent tissues. Similarly, kidney cancer studies from Gumz [15] and Yusenko [16] confirmed that GPX1 was expressed higher in ccRCC tissues than that in adjacent normal tissues (Figure 2B–2C). Next, we evaluated the expression levels of GPX1 and its association with clinicopathological parameters in ccRCC patients based on age, gender, T stage, N stage, M stage, Grade classification, histopathological stage (Figure 2D–2I, Table 1). The evaluation found that the high expression of GPX1 was positively correlated with the ccRCC stage. These results indicate that GPX1 is overexpressed in ccRCC and positively correlated with higher tumor stage.

**Table 1:** The correlation between GPX1 expression and clinicopathological parameters of ccRCC patients

**Parameter**		**Number**	**GPX1 mRNA expression**		**P value**
			**Low (n=263)**	**High (n=263)**	
Age (years)	<= 60	261	135	126	
	>60	265	128	137	0.433
Gender	Male	341	153	188	
	Female	185	110	75	0.001
T stage	T1+T2	337	188	149	
	T3+T4	189	75	114	0.000
N stage	N0+NX	510	260	250	
	N1	16	3	13	0.011
M stage	M0+MX	448	236	212	
	M1	78	27	51	0.003
G stage	G1+G2+GX	246	149	97	
	G3+G4	280	114	166	0.000
Pathologic stage	Stage I+II	319	183	136	
	Stage III+IV	207	80	127	0.000

### The diagnostic value of high GPX1 expression in ccRCC patients

**Figure 3:**
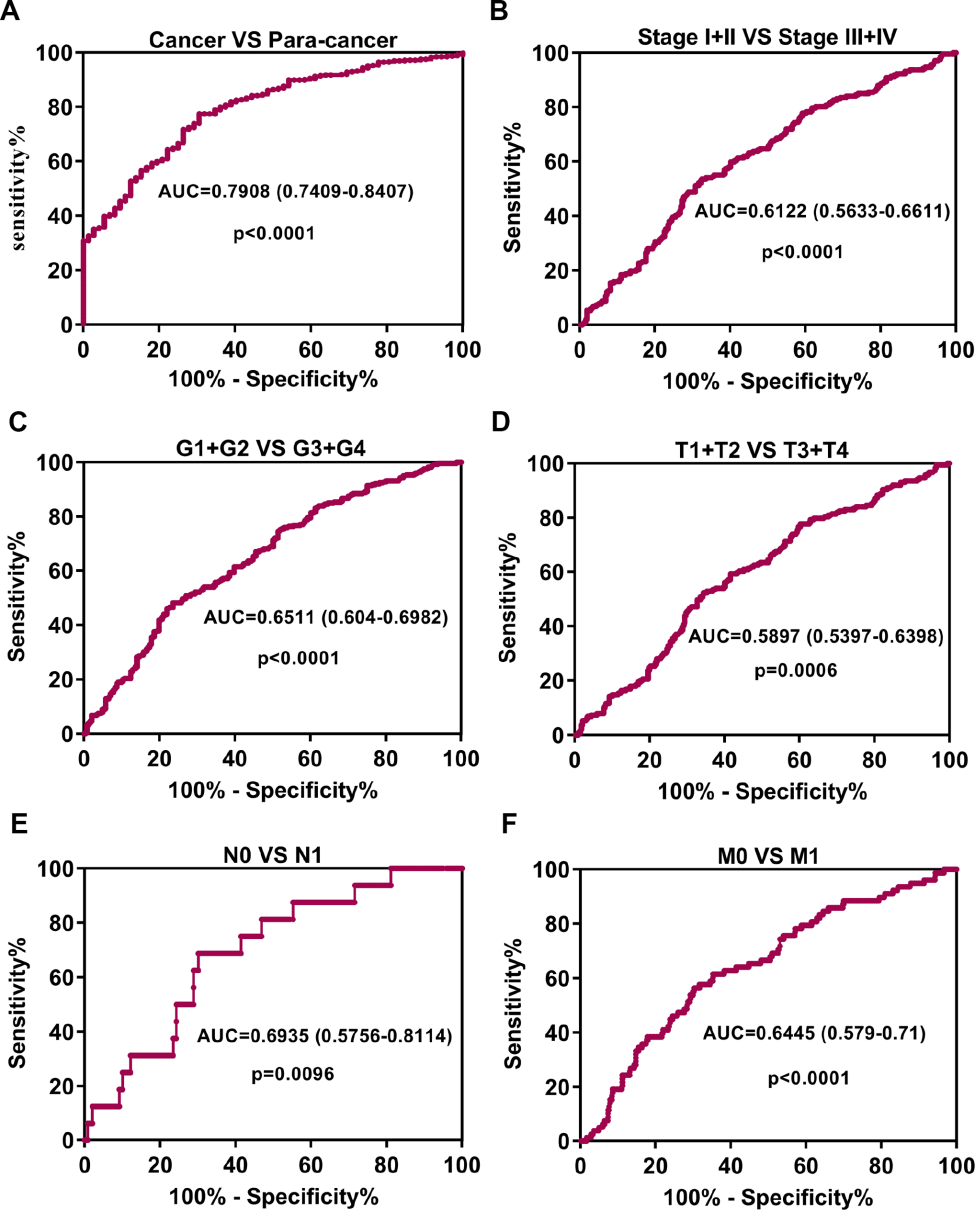
**Up-regulation of GPX1 may serve as a potential diagnostic biomarker in ccRCC.** (**A**) ROC curve analysis indicated that GPX1 could efficiently distinguish ccRCC from normal individual. The area under curve (AUC) was 0.7908 (p<0.0001). ROC curve analysis towards the expression levels of GPX1 mRNA in ccRCC subgroups against pathological stage (**B**), G stage (**C**), T stage (**D**), N stage (**E**) and M stage (**F**).

To probe whether the high expression of GPX1 possesses diagnostic significance in ccRCC patients, the ROC curves were used to analyze the diagnostic value of high GPX1 expression in various clinicopathological parameters from TCGA-KIRC datasets. ROC curve analysis showed that GPX1 could statistically distinguish ccRCC from normal tissue producing an area under the curve (AUC) of 0.7908 (95% CI: 0.7409-0.8407; p < 0.0001). Moreover, we performed a ROC curve analysis in the subgroup of ccRCC patients against pathological stage, T stage, N stage, M stage and G stage. Subgroup ROC curve analysis implied that the high expression of GPX1 might have diagnostic value for ccRCC patients with pathological stage (I + II) / (III + IV) (Figure 3B, AUC = 0.6122, p < 0.0001), (G1 + G2) / (G3 + G4) stage (Figure 3C, AUC = 0.6511, p < 0.0001), (T1 + T2) / (T3 + T4) (Figure 3D, AUC = 0.5897, p = 0.0006), N0 / N1 stage (Figure 3E, AUC = 0.6935, p = 0.0096), M0 / M1 stage (Figure 3F, AUC = 0.6445, p < 0.0001). These results implicitly suggested that GPX1 might have diagnostic value for patients with ccRCC.

### The prognostic value of high GPX1 expression in ccRCC patients

**Figure 4:**
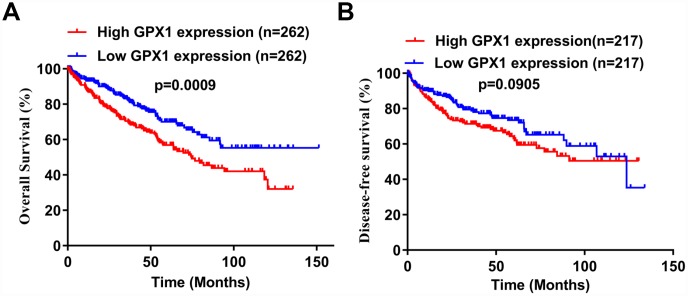
**Potential prognostic value of high GPX1 expression in ccRCC patients.** (**A**–**B**) Based on the median value of GPX1 expression, ccRCC samples from the TCGA database were divided into high GPX1 expression group and low GPX1 expression group. Kaplan-Meier curves were used to analyze the correlation between GPX1 levels and overall survival time (OS) and disease-free survival time (DFS) in ccRCC samples.

To investigate whether high expression of GPX1 is associated with prognosis in patients with ccRCC, we performed Kaplan-Meier analysis using data from the TCGA-KIRC database. The analysis found that high GPX1 expression predicted a worse overall survival (OS) in ccRCC patients (Figure 4A), however, GPX1 expression does not affect disease-free survival (DFS) (Figure 4B).

### The expression levels of GPX1 were confirmed in RCC cell lines and tissues

**Figure 5:**
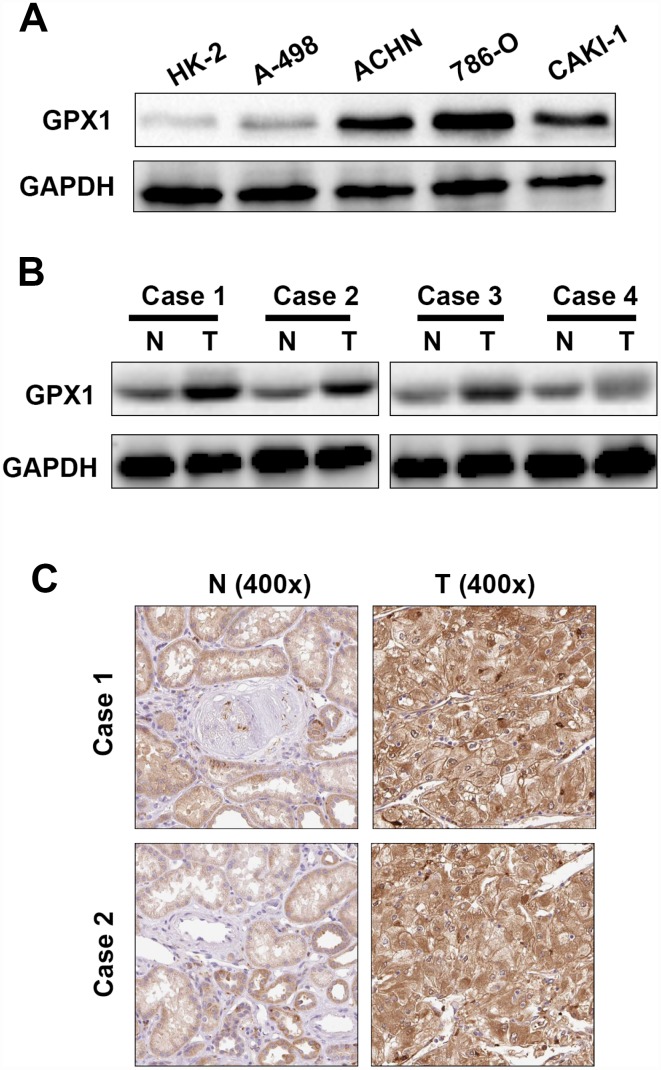
**The expression levels of GPX1 in renal cancer cells and tissues.** (**A**) Western blotting analysis of GPX1 expression levels in renal cancer cell lines (786-O, ACHN, A-498, Caki-1) and renal normal epithelial cells (HK-2). (**B**) Western blotting analysis of GPX1 expression levels in 4 pairs of ccRCC tissues (T = tumor tissue, N = normal tissue). (**C**) Immunohistochemical analysis of GPX1 expression levels in normal renal tissues and ccRCC tissues (T = tumor tissue, N = normal tissue).

To further verify the results of the ONCOMINE and TCGA databases, GPX1 was subjected to immunoblotting in RCC cell lines and tissues. As shown in Figure 5A, 5B, the expression levels of GPX1 in RCC cell lines were significantly higher than that of normal renal epithelial cell HK-2, and the expression of GPX1 in RCC tissues was also obviously higher than that in adjacent normal tissues. The expression levels of GPX1 were also detected by immunohistochemistry (IHC) in paired RCC tissues, and the IHC results were consistent with the results of immunoblotting (Figure 5C). The above results indicate that GPX1 is up-regulated in RCC cell lines and tissues, consistent with TCGA and ONCOMINE database results.

### Knockdown of GPX1 expression level inhibits proliferation of renal cancer cells in vitro

**Figure 6:**
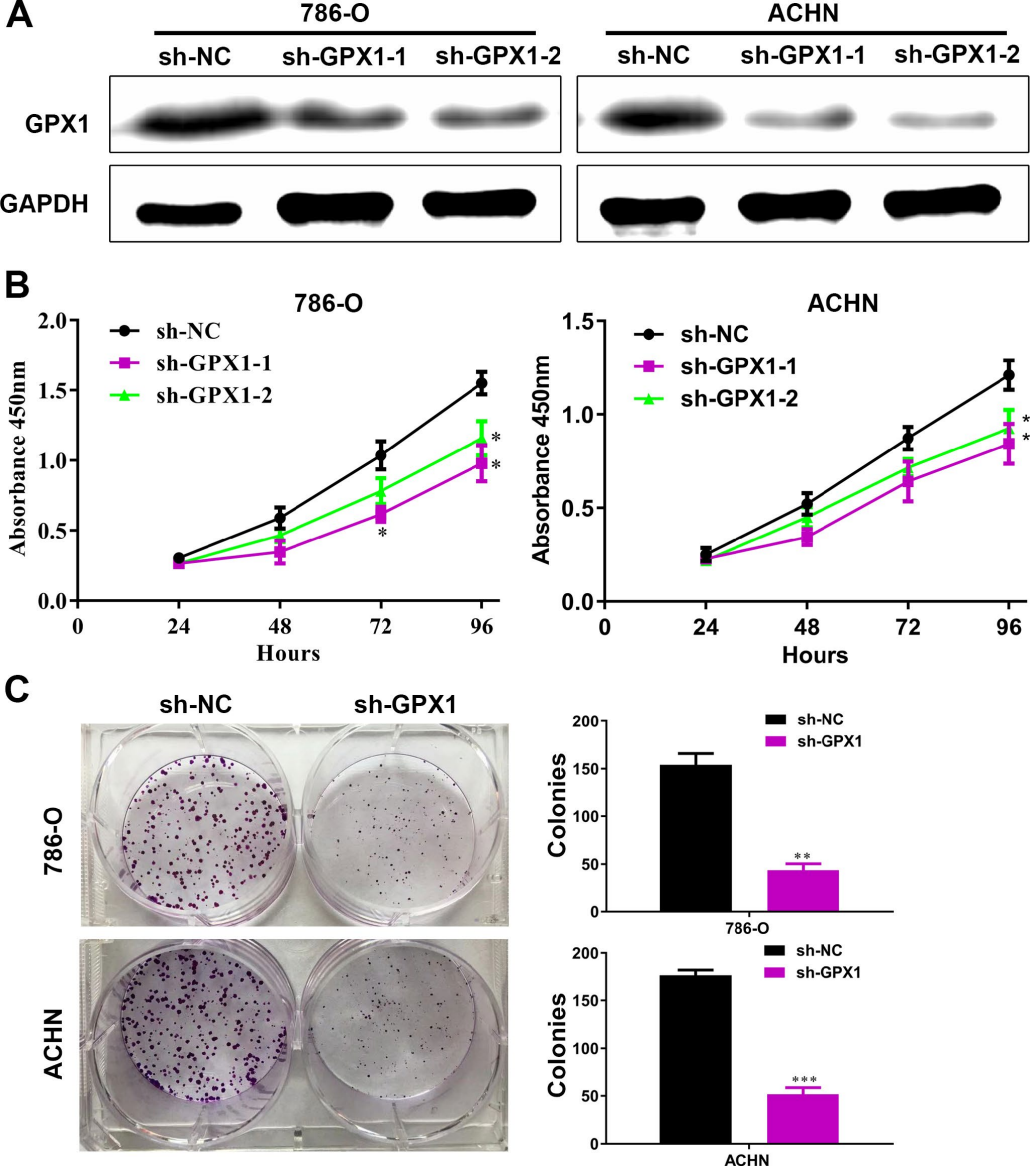
**The knockdown of GPX1 levels inhibits the proliferation capability of renal cancer cells *in vitro*.** (**A**) As shown by immunoblotting results, the expression levels of GPX1 were knocked down by transfecting sh-GPX1 plasmids. (**B**) CCK8 results showed that GPX1 knockdown significantly inhibited the proliferation of renal cancer cells. (**C**) GPX1 knockdown obviously reduced the colony formation ability of renal cancer cells. (***, P < 0.001, **, P < 0.01, *, P < 0.05, compared with the respective control).

To explore the function of GPX1 in renal cancer, we first examined the effect of GPX1 on the growth of renal cancer cells in vitro, a key factor in tumor volume enlargement. Considering the high expression level of GPX1 in detecting renal cancer cell lines, we used the sh-RNA knockdown method. Two sh-GPX1 knockdown plasmids and corresponding sh-NC plasmids were transfected into 786-O and ACHN cells, resulting in consistent GPX1 knockdown (Figure 6A). CCK8 assays show that knockdown of GPX1 can inhibit the proliferation of renal cancer cells (Figure 6B). Colony formation assays reveal that knockdown of GPX1 can reduce clonogenic capacity of renal cancer cells (Figure 6C). These results reversely confirm that GPX1 can promote the growth of renal cancer cells.

### Knockdown of GPX1 expression level inhibits migration and invasion of renal cancer cells in vitro

**Figure 7:**
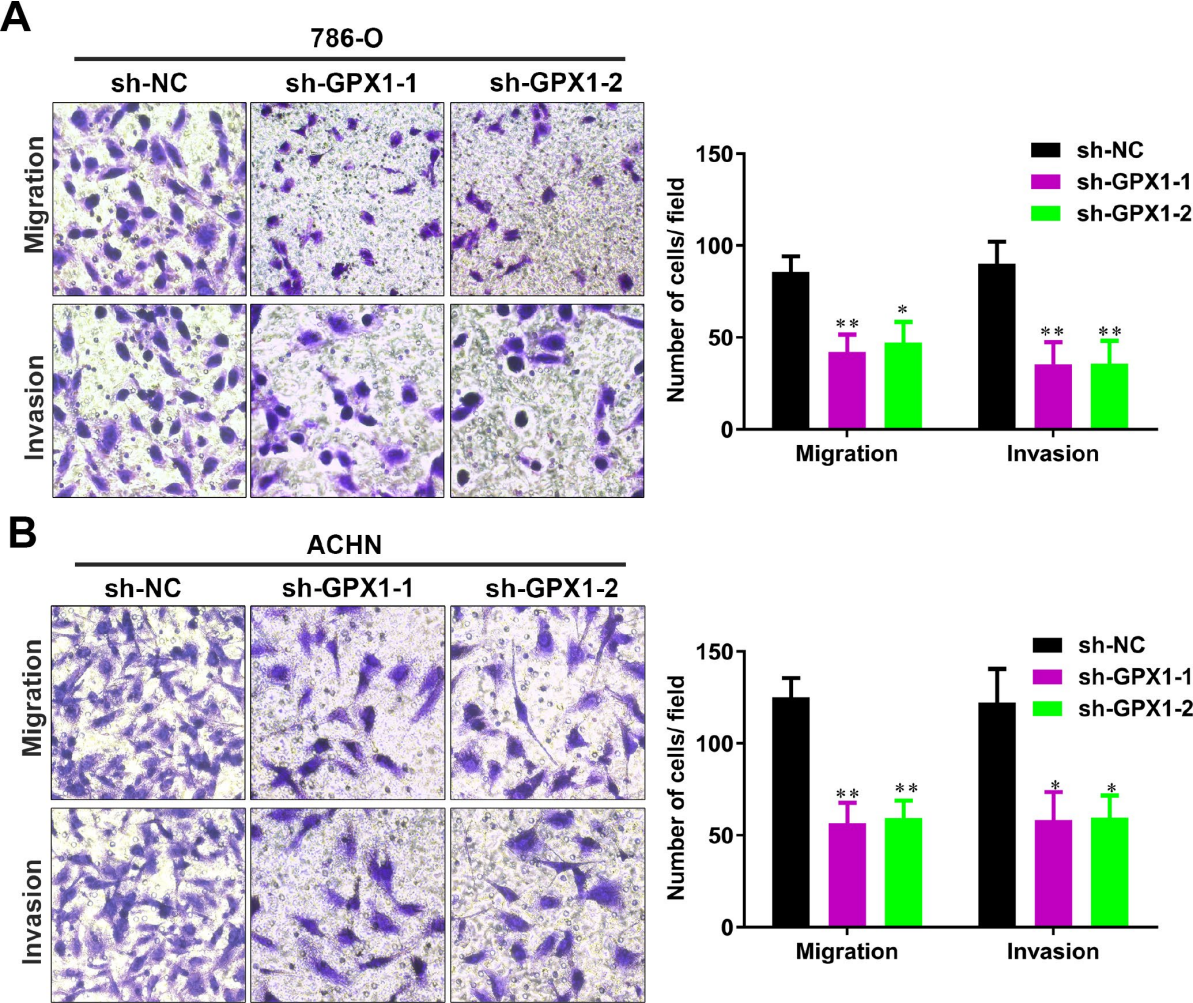
**The knockdown of GPX1 levels significantly inhibits migration and invasion of renal cancer cells *in vitro*.** (**A**, **B**) Transwell assays analysis of the impact of GPX1 levels knockdown on cell migration and invasion of 786-O and ACHN. (**, P < 0.01, *, P < 0.05, compared with the respective control).

To investigate whether GPX1 affects the migration and invasion of renal cancer cells, we performed the transwell assays. As shown in Figure 7A-B, knockdown of GPX1 significantly reduced the migration and invasion capability of 786-O and ACHN cells. These results reversely suggest that GPX1 promotes migration and invasion of renal cancer cells.

## DISCUSSION

Renal cell carcinoma is the most common upper urinary tract tumor in the urinary system. About 15% of RCC patients are metastatic at the time of diagnosis [4]. Therefore, early screening of these patients with metastatic RCC (mRCC) is beneficial to the treatment and prognosis of patients. However, there is currently no effective biomarker for early diagnosis of RCC in clinical treatment, and the molecular mechanism of RCC metastasis remains unclear.

Reactive oxygen species (ROS) can be produced by normal cells and tumor cells during normal oxidative respiration and, if not cleared timely by the antioxidant enzyme system, can cause oxidative stress damage to DNA, proteins and organelles [5]. In cells, ROS are mainly produced by redox-reactive enzymes, such as NADPH oxidase subtypes and cytochrome P-450 subtypes, and mitochondrial respiration in the form of superoxide [17, 18]. In normal cells, ROS are in a balance between formation and clearance, while ROS in tumor cells are in a state of accumulation due to mitochondrial dysfunction and high metabolic status [7]. Cumulative ROS levels have been reported to be associated with tumor initiation, tumor transformation, and chemotherapy tolerance in tumors [19, 20]. However, accumulated ROS can be detoxified by anti-peroxidase system in tumor cells to avoid oxidative stress damage [21]. The efficacy of ROS and antioxidant enzyme systems in tumors is extremely complex and sometimes contradictory. Antioxidant enzymes were initially thought to have a therapeutic effect on tumors by eliminating ROS-mediated tumorigenesis and progression [22–24]. However, several studies have confirmed that antioxidant enzymes have no benefit for tumor therapy or that antioxidant enzymes promote tumor progression [25–27].

GPX1, a member of the glutathione peroxidases (GPXs) family, was ubiquitously distributed in mitochondria and cytoplasm. GPX1 has been reported by several research groups to play opposite roles in different tumors. Such as, the expression levels of GPX1 were highly expressed and predicted poor prognosis in laryngeal squamous cell carcinoma [28]. Increased expression of GPX1 promotes cell proliferation, migration, invasion, and cisplatin-resistance in esophageal squamous cell carcinoma [29]. Overexpression of GPX1 increased the number of tumors and promotes their growth in a mouse model of skin cancer [12]. In summary, GPX1 may play different roles in different tumors. However, few studies have reported the expression and roles of GPX1 in renal cell carcinoma.

In this study, we first analyzed the expression levels of GPXs family members in ccRCC using the TCGA cancer database. Unexpectedly, we found that among the 8 GPXs, only GPX1 was up-regulated in ccRCC. So, we focused our research on GPX1. ONCOMINE database was used to detect the reliability of the TCGA database. Gumz studies and Yusenko studies from the ONCOMINE database confirmed the up-regulation of GPX1 expression in ccRCC. Bioinformatics analysis found that high expression of GPX1 was positively correlated with tumor stage, distant metastasis and lymphatic metastasis. ROC curve analysis found that high expression of GPX1 could effectively distinguish ccRCC from normal individuals. Moreover, enhanced expression of GPX1 predicted a worse overall survival in ccRCC patients. To confirm the accuracy of the cancer database, we examined the expression of GPX1 in RCC cells and tissues, and the results were consistent with the database predictions. In terms of function, GPX1 promotes proliferation, colony formation capacity, migration and invasion of renal cancer cells. However, our research also has some shortcomings, that is, the mechanism of GPX1 overexpression and molecular mechanisms of GPX1-promoting renal cell carcinoma progression remain unclear. Moreover, we only have one normal renal epithelial cell line (HK-2) as a control cell line. We will continue to explore these potential molecular mechanisms in subsequent studies, aiming to provide a theoretical basis for GPX1-targeted therapy in RCC patients.

In summary, we confirm the high expression of GPX1 promoted the progression in renal cell carcinoma. Moreover, GPX1 has the potential to be a promising biomarker for the diagnosis and prognosis of ccRCC patients. In addition, targeting GPX1 may provide new directions and strategies for mRCC treatment.

## MATERIALS AND METHODS

### Cell culture

The human RCC cell lines 786-O, A498, ACHN, Caki-1 and normal renal tubular epithelial cells HK-2 were purchased from ATCC. OS-RC-2 cell line was a gift from the Department of Urology of Wuhan Tongji Hospital. All cells were cultured in DMEM medium containing 10% FBS and 1% penicillin-streptomycin.

### Cell transfection

Short hairpin RNA (sh-RNA) against GPX1 and corresponding negative control (sh-NC) were constructed by Vigene Biosciences (Shan Dong, China). The sh-RNA sequences are subjected to BLAST to avoid off-target effects. Constructed sh-RNA sequences and sh-NC sequences were transferred into cells using Lipofectamine 2000 reagent. The sh-RNA sequences were listed as following: sh-GPX1-1: 5′-GCTTCCAGACCATTGACA TCG-3′, sh-GPX1-2: 5′-ACCATTGACATCGAGCCTG AC-3′, sh-NC: 5′-UUCUCCGAACGUGUCACGUTT-3′,

### CCK8 proliferation assays and colony formation assays

CCK8 (Beyotime Institute of Biotechnology) assay was used to test the proliferative capacity of tumor cells. 786-O (sh-GPX1 and sh-NC) and ACHN (sh-GPX1 and sh-NC) were seeded into 96-well plates with 1000 cells per well. The absorbance of each well was detected by a microplate reader at a wavelength of 450 nm.

As for the colony formation assays, 786-O (sh-GPX1 and sh-NC) and ACHN (sh-GPX1 and sh-NC) were seeded into 6-well plates with 1000 cells per well. After 2 weeks of growth, these cell colonies were fixed by methanol and then stained with crystal violet.

### Immunohistochemistry assay

Briefly, RCC tissues and adjacent normal tissues were fixed by using 10% formalin. Then, those tissues were dehydrated, and embedded in paraffin. Those tissue sections were incubated with GPX1 rabbit polyclonal antibody (1:100, ABclonal, Wuhan, China) overnight. After washing with PBS thrice, those sections were incubated with secondary antibodies conjugated to horseradish peroxidase labeled polymers. Finally, those sections were counterstained with hematoxylin.

### Transwell migration and invasion experiments

These experiments were done as previously described [30].

### Western blotting experiments

RCC Tissues or cells were lysed in RIPA lysis buffer containing a protease inhibitor cocktail tablet (Roche) and 1 mM Phenylmethylsulfonyl fluoride (PMSF). The western blotting experiments were done as previously described [31].

### Patient kidney cancer tissue samples

We collected RCC tissues and adjacent normal tissues in 50 case patients who were subjected to nephrectomies or partial nephrectomies operated at Wuhan Union Hospital between 2016 and 2018. The characteristics of 50 patients with ccRCC are summarized in Table 2. The collected ccRCC tissues were divided into two parts: 20 pairs were promptly frozen in liquid nitrogen for western blot assays; the other 30 pairs were fixed with formalin and paraffin-embedded for matched immunohistochemistry analysis. Before surgery, these patients did not receive any anti-tumor therapy, including chemotherapy, radiotherapy or targeted therapy. We have signed informed consent form with all patients. This study was approved by the Ethics Committee of Huazhong University of Science and Technology.

**Table 2:** The ccRCC patient characteristics in this study (n=50, 2016-2018).

**Characteristic**	**N (%)**
Age	
Mean ± SEM (years)	55 ± 13
Gender	
Male/female	35/15
Tumor size	
Mean ± SEM (cm)	5.2 ± 2.8
Location of cancer	
Right/left	28/22
T stage	
pT1a	16 (32)
pT1b	15 (30)
pT2a	8 (16)
pT2b	4 (8)
pT3	4 (8)
pT4	3 (6)
N stage	
N0	46 (92)
N1	4(8)
M stage	
M0	48 (96)
M1	2 (4)
Fuhrman grade	
1	23 (46)
2	20 (40)
3	5 (10)
4	2 (4)

### Cancer database bioinformatic analysis

The mRNA expression level data of GPX1 in ccRCC tissues and corresponding normal tissue and clinicopathological parameters data including patient gender, age, T stage, M stage, N stage, Grade stage, histopathological stage, overall survival (OS), disease-free survival (DFS) were downloaded from the TCGA-KIRC dataset. The mRNA expression level data of GPX1 were also downloaded from the ONCOMINE database. Kaplan-Meier curves and ROC curves were analyzed using mRNA levels from TCGA-KIRC dataset.

### Statistical analysis

SPSS statistical software and Graphpad Prism 7.0 were used for statistical analysis. The GPX1 mRNA levels were analyzed in different clinicopathological parameters of ccRCC using the Mann-Whitney test. Pearson's chi-square test was used to analyze the correlation between GPX1 expression levels and clinicopathological parameters of ccRCC. The receiver operating characteristic (ROC) curve was used to analyze the expression level of GPX1 to distinguish ccRCC patients and obtain the area under the curve. The Kaplan-Meier curve was used to analyze the relationship between the expression level of GPX1 and the overall survival and progression-free survival of ccRCC patients. Each group of data is presented as mean ± SD. The p value < 0.05 of statistical analysis was considered to have significant differences.
